# Enhanced cellular engraftment of adipose-derived mesenchymal stem cell spheroids by using nanosheets as scaffolds

**DOI:** 10.1038/s41598-021-93642-6

**Published:** 2021-07-14

**Authors:** Hisato Nagano, Yoshitaka Suematsu, Megumi Takuma, Shimpo Aoki, Ayano Satoh, Eiji Takayama, Manabu Kinoshita, Yuji Morimoto, Shinji Takeoka, Toshinori Fujie, Tomoharu Kiyosawa

**Affiliations:** 1 Department of Plastic and Reconstructive Surgery, National Defense Medical College, Tokorozawa, Saitama 359-8513 Japan; 2 Department of Life Science and Medical Bioscience, Graduate School of Advanced Science and Engineering, Waseda University, Tokyo, 162-8480 Japan; 3 School of Life Science and Technology, Tokyo Institute of Technology, Yokohama, Kanagawa 226-8501 Japan; 4 Tissue Engineering and Wound Healing Laboratory, Division of Plastic Surgery, Brigham and Women’s Hospital, Harvard Medical School, 75 Francis Street, Boston, MA 02115 USA; 5 Graduate School of Interdisciplinary Science and Engineering in Health Systems, Okayama University, Okayama, 700-0082 Japan; 6 Department of Oral Biochemistry, Asahi University School of Dentistry, Gifu, 501-0296 Japan; 7 Department of Immunology and Microbiology, National Defense Medical College, Tokorozawa, Saitama 359-8513 Japan; 8 Department of Physiology, National Defense Medical College, Tokorozawa, Saitama 359-8513 Japan; 9 Institute for Advanced Research of Biosystem Dynamics, Research Institute for Science and Engineering, Waseda University, 3-4-1 Ohkubo, Shinjuku-ku, Tokyo 169-8555 Japan

**Keywords:** Biomaterials - cells, Stem-cell therapies

## Abstract

The short survival time of transplanted adipose-derived mesenchymal stem cells (ASCs) is a problem for skin wound healing. Transplantation after the formation of cellular spheroids has been investigated as a promising method for prolonging cellular survival. However, there have been technical restrictions for transplantation of spheroids in clinical practice. Here, we show an effective method for transplantation of ASC spheroids onto skin wounds in order to efficiently cure refractory ulcers. To assist anchoring of spheroids onto skin wounds, we used a 120-nm-thick free-standing film (nanosheet) that has a highly adhesive property. Bioluminescence imaging showed that ASC spheroids carried by the nanosheet survived for 14 days, which is about two-times longer than that previously reported. Wounds treated with a nanosheet carrying ASC spheroids were 4-times smaller than untreated wounds on day 14. This method for transplantation of spheroids could be applied to cell therapy for various refractory skin wounds.

## Introduction

Transplantation of adipose-derived mesenchymal stem cells (ASCs) is an attractive therapy for refractory skin ulcers such as diabetic wounds, critical limb ischemia, radiation ulcers and ulcers caused by extravasation of cytotoxic drugs. However, the short survival time of ASCs after transplantation is a major problem that hampers the therapeutic efficiency of ASCs^1–3^. In previous studies, most of the transplanted cells were lost within 72 h after transplantation^1, 4^. The short survival time is thought to be caused by a low level of nutrition, local hypoxia^5, 6^ and anoikis-mediated cell death, which is an adherent cell apoptosis due to the loss of attachment between cells and the extracellular matrix (ECM)^7^.

A cluster formed by aggregated multiple cells is called a spheroid, in which cell–cell and cell-ECM interactions are preserved. In recent years, stem cell spheroids have been receiving much attention because they may prolong cellular survival^8, 9^. Due to the cell–cell and cell–ECM interactions, a spheroid has the resistance to anoikis and harsh microenvironments^10, 11^. Additionally, spheroids enhance a paracrine effect^12, 13^ compared to dissociated cells that do not form spheroids. Accordingly, transplantation of spheroids onto a skin wound is a more promising method than transplantation of dissociated cells^14, 15^. This was also confirmed by Amos et al. using ASC spheroids. On the other hand, in their report, the case of using smaller number of ASCs spheroids did not have a significant effect on wound healing compared to the control group^16^. This observation suggests that it is important for spheroids to remain on the wound in spheroid transplantation. In other words, prolongation of the activities of transplanted spheroids is the most likely key to success of skin wound healing. We considered that the use of a scaffold that holds spheroids onto the wound is essential for prolonging the activities of spheroids. Several materials (coatings^17^, hydrogels^18, 19^, other materials^20, 21^) have been used to support ASC spheroid formation and retention. Although there have been a few reports on the use of scaffolds for transplantation of stem cell spheroids onto skin wounds^18–20^, insufficient attachment between cells and tissues could limit the engraftment efficiency of ASC transplantation. It has not been determined whether the use of a scaffold prolongs the activities of spheroids and enhances skin wound healing.

A nanosheet made of poly(d,l-lactic acid) (PDLLA), which is a free-standing thin film (thickness: < 1 μm) that we developed, was used as a scaffold in this study. PDLLA is known to be a biocompatible and biodegradable polymer. In addition, PDLLA is soluble in ethyl acetate and the polymer concentration can thus be conveniently adjusted, and it is benign to the wet film coating process such as gravure coating to control the film thickness at a nanometer scale^22^. The nanosheet has strong physical adsorption via Van der Waals forces due to its thinness^23^. Hence, a nanosheet can be an excellent scaffold that carries ASCs onto the wound without the need for an adhesive agent^24^. Nishiwaki et al. reported the wound healing effect of ASC transplantation with a tri-layered porous nanosheet. However, further improvement of the wound healing effect was considered to be difficult because transplanted ASCs took a two-dimensional monolayer state.

The aim of this study was to establish a method for transplantation of ASC spheroids by using a nanosheet as a scaffold in order to efficiently cure a refractory skin ulcer. We hypothesized that (1) ASC spheroids carried by a nanosheet would survive on the skin wound surface and that the nanosheet would protect the transplanted ASC spheroids from the outer environment and (2) the transplanted ASC spheroids would promote wound healing. In this study, we used a model of refractory skin ulcer induced by mitomycin C because we needed a skin ulcer that does not heal for at least two weeks for long-term cell tracking.

## Results

**Figure 1:**
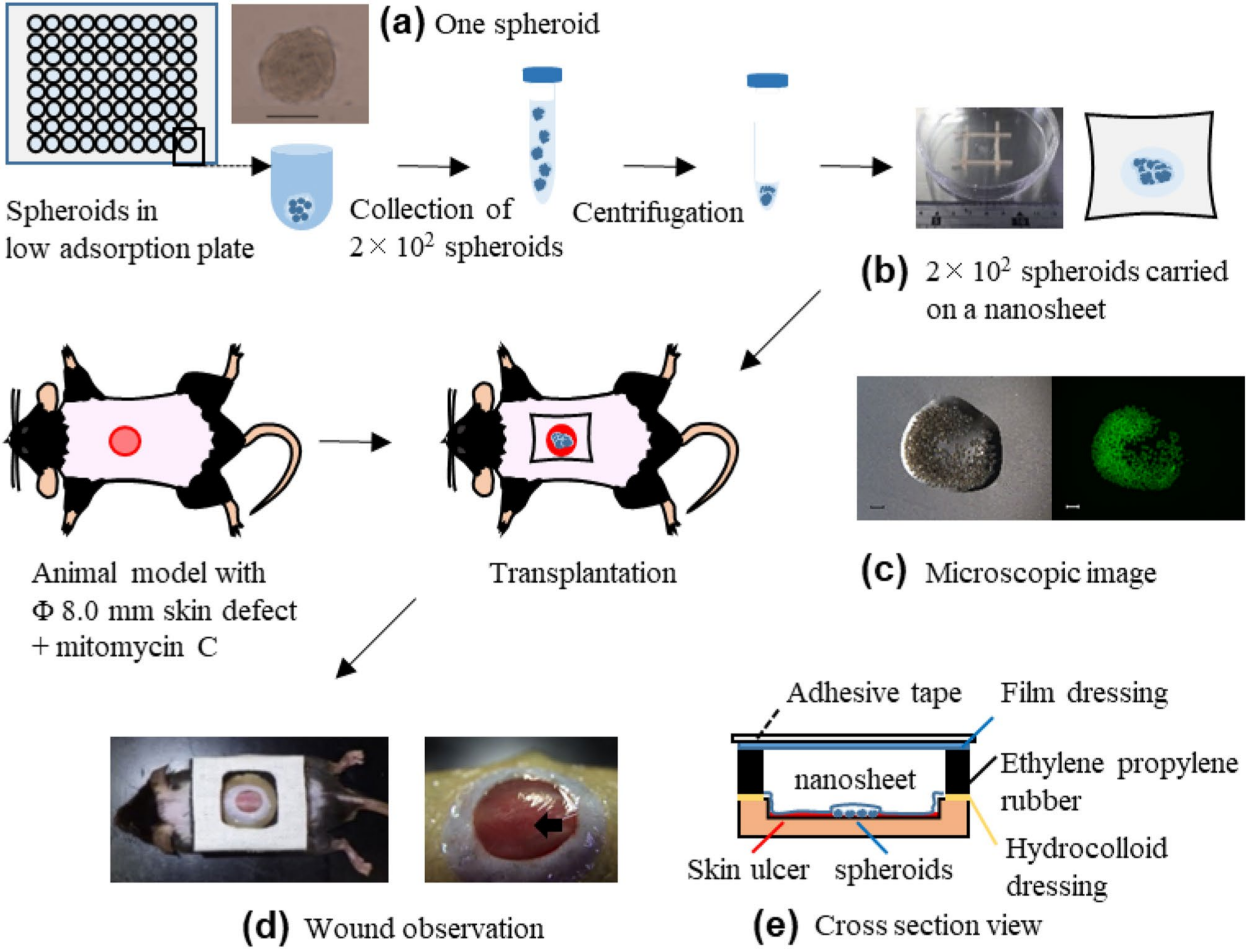
Outline of making animal models. (**a**) One spheroid consisting of 1 × 10^3^ ASCs was formed in a well of a low adsorption plate. Two hundred ASC spheroids were then collected into a conical tube and centrifugated. Scale bar = 100 μm. (**b**) Concentrated 2 × 10^2^ spheroids were resuspended in Hanks’ balanced salt solution and were gently pipetted onto the center of a pristine nanosheet with a size of 3 × 3 cm. Spheroids carried by the nanosheet were engrafted onto the refractory skin ulcer. (**c**) *Left*: bright-field microscopy image of spheroids carried by the nanosheet. (**c**) *Right*: fluorescence image. (**d**) *Left*: configuration of the wound dressing is shown. The wound was surrounded by hydrocolloid dressing (Yellow) and ethylene/propylene rubber (Black). The top was covered with a film dressing (Blue) and adhesive tape (White). (**d**) *Right*: spheroids could be observed by the naked eye through the nanosheet just after transplantation (black arrow). (**e**) Gross image of the wound after transplantation followed by several processes of dressing.

For the development of skin wounds, we used a refractory skin ulcer mouse model that was previously reported^25^. In brief, the skin and subcutaneous tissue in a partial region of the back area of each mouse were removed, and mitomycin C was topically applied onto the exposed fascia. The animal model showed suppression of wound contracture and granulation formation and, as a result, the wound did not heal for at least two weeks. ASCs were formed into a spheroid using a low adsorption plate (Fig. 1a). ASC spheroids carried by the nanosheet (Fig. 1b,c) were then transplanted onto the lesion (Fig. 1d,e).

### Prolonged cellular survival time of ASC spheroids

**Figure 2:**
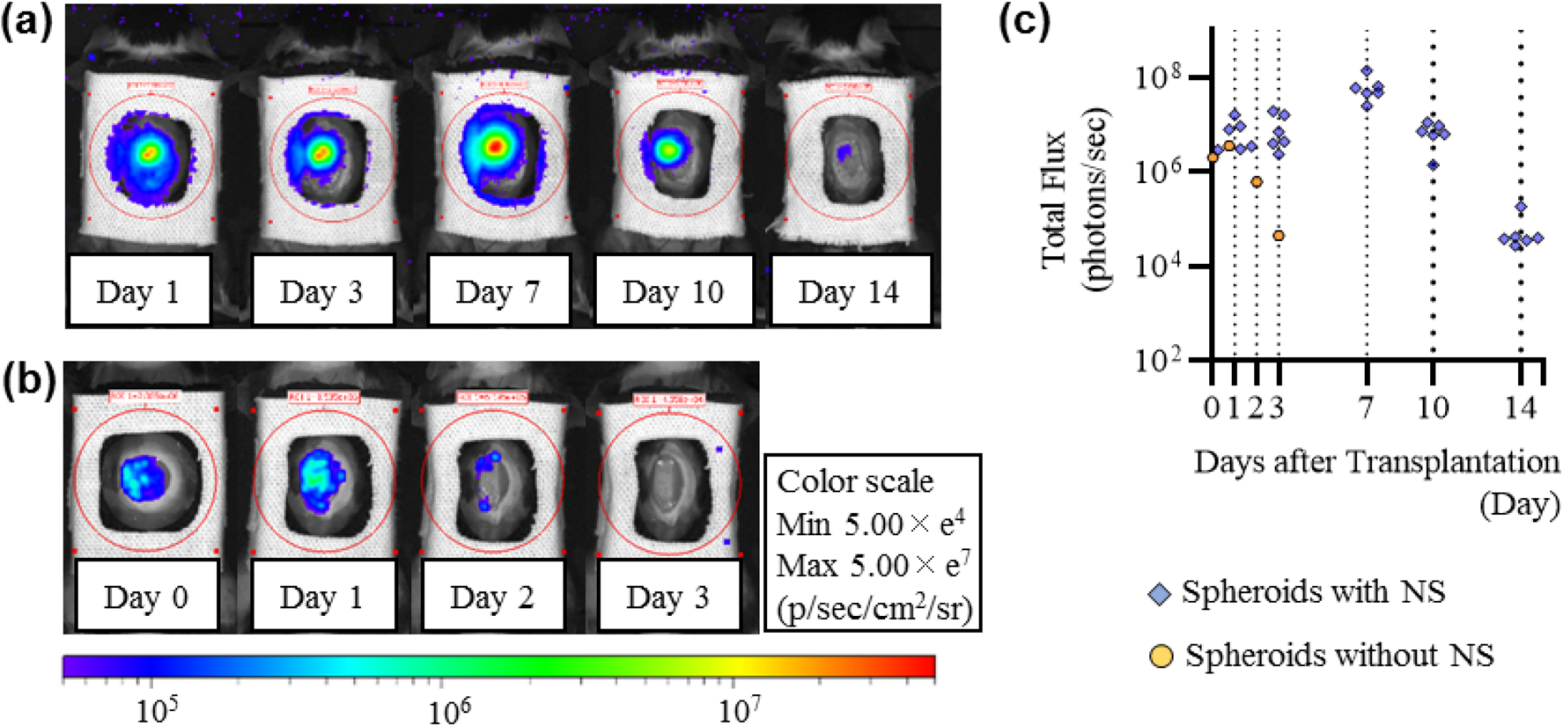
Images of luminescence from ASC spheroids. (**a**) Time-lapse images of luminescence from ASC spheroids carried by the nanosheet (representative images). The luminescence peaked at day 7 and was observed for 14 days. (**b**) Time-lapse images of luminescence from ASC spheroids that were simply dropped onto the wound (no nanosheet) (representative images). The luminescence decreased gradually and was observed for only 3 days at most. (**c**) Transition of intensity of luminescence from ASC spheroids carried by the nanosheet (*blue rhombuses*) and ASC spheroids without a nanosheet (*yellow circles*) after transplantation.

Confirmation of cellular engraftment and quantitative measurement of the viability of ASC spheroids were carried out using a noninvasive bioluminescence imaging (BLI) system (IVIS, PerkinElmer). To visualize the ASCs, we used luminescent ASCs (nano-lantern ASCs) that had been transfected with a luciferase gene, nano-lantern, using a retrovirus. The luminescence intensity of nano-lantern ASCs increased with increase in the number of spheroids (Supplementary Fig. 1). There is no significant difference between the phenotype of ASCs and that of nano-lantern ASCs (Supplementary Fig. 2). When the nano-lantern ASC spheroids carried by the nanosheet were transplanted onto the wound area, luminescence from the nano-lantern ASCs spheroids was observed for up to 14 days at maximum (Fig. 2a). On the other hand, when nano-lantern ASC spheroids alone without a nanosheet were transplanted, luminescence of the nano-lantern ASC spheroids disappeared in a few days (Fig. 2b). In more detail, when the nanosheet was used, luminescence from the nano-lantern ASC spheroids was confirmed for at least 10 days in all of the treated mice. The intensity of the luminescence peaked on day 7: the intensity on day 7 was 10-times greater than the intensities on other days (Fig. 2c).

### Characterization of PDLLA nanosheets

**Figure 3:**
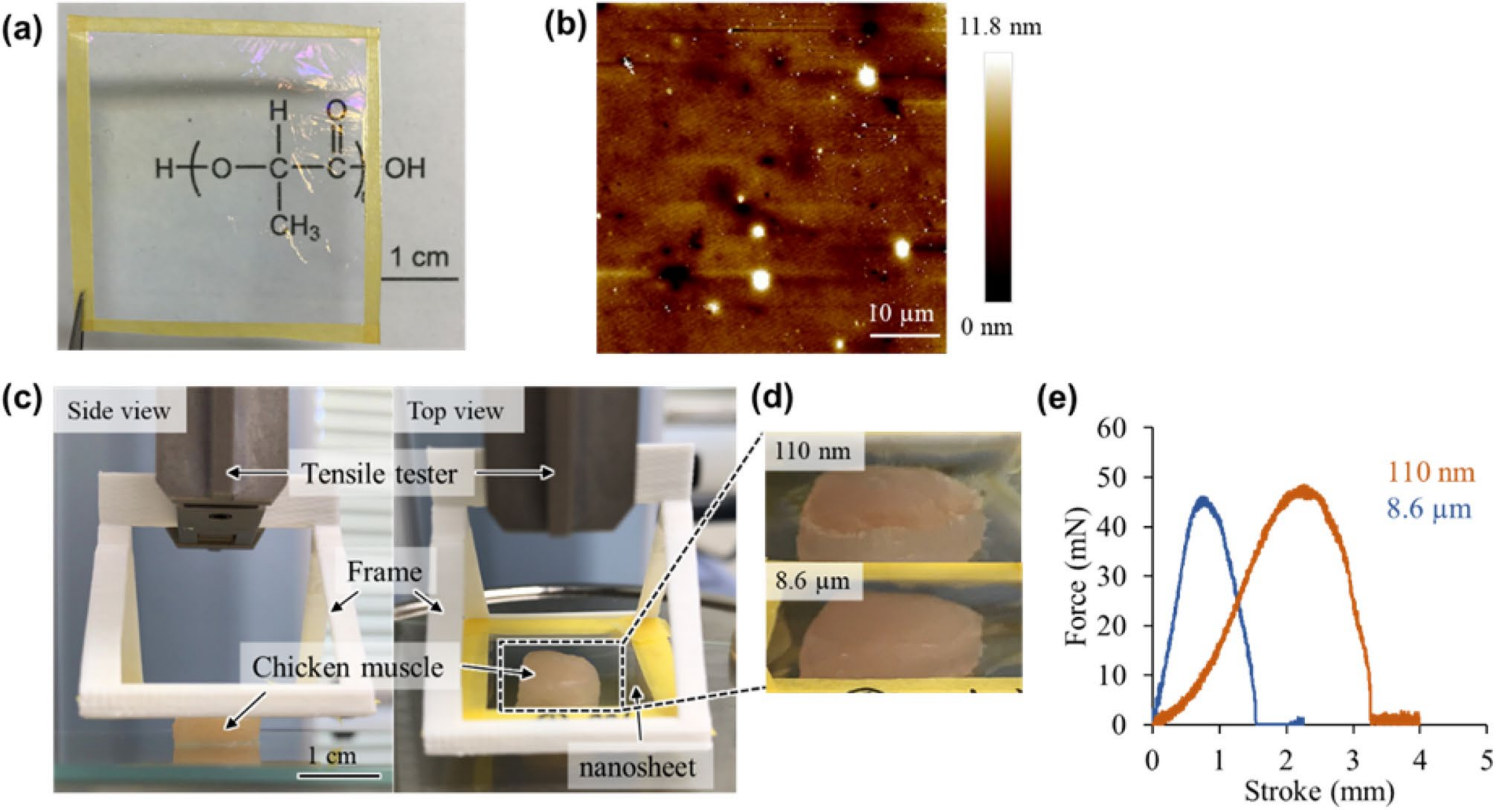
Optical, morphological and mechanical characterization of PDLLA nanosheets. (**a**) An optical image of PDLLA nanosheet suspended by a tape frame. (**b**) AFM height image of PDLLA nanosheet. (**c**) Experimental setup of tack-separation test; PDLLA nanosheet supported by the 3D-printed plastic frame interfacing biological tissue (i.e., chicken muscle), which was pulled by tensile tester. (**d**) Optical images of PDLLA thin films with different thickness (110 nm and 8.6 µm), conforming to the chicken muscle. (**e**) Force–stroke curves of different PDLLA thin films examined by the tack-separation test.

We prepared PDLLA nanosheets by a gravure-printing-based roll-to-roll process. The fabricated nanosheet (thickness: 110 nm) was transparent (Fig. 3a) with a smooth surface (root-mean-square roughness: 1.87 nm) as shown by atomic force microscopy (AFM) (Fig. 3b). We also evaluated the adhesive property of PDLLA nanosheets by a tack-separation test using a tensile tester^26^ (Fig. 3c). We investigated two types of PDLLA thin films with different thicknesses (110 nm and 8.6 µm) and compared their degrees of conformability (represented by stroke) and adhesiveness (represented by force) to biological tissue such as chicken muscle. The 110-nm-thick nanosheet showed a higher degree of conformability than that of the 8.6-µm-thick sheet to the chicken muscle surface (Fig. 3d), resulting in a longer separation stroke (Fig. 3e). These results indicate that the PDLLA nanosheet adhered well to a wound surface due to its flexible structure.

**Figure 4:**
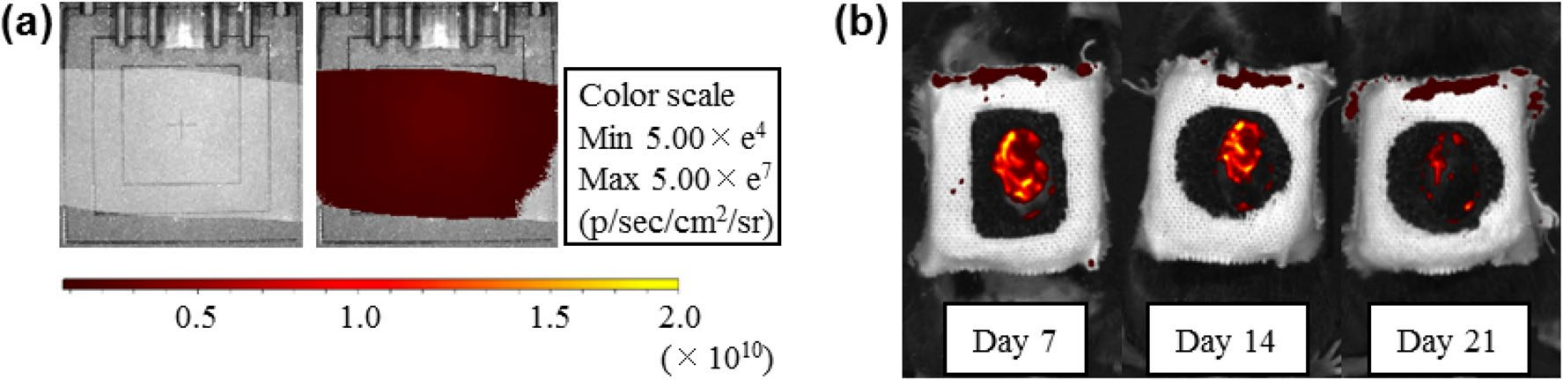
IVIS image of Nile Red nanosheet. (**a**) A nanosheet containing Nile Red was made to observe transition of the adhesion state of the nanosheet on the wound. (**a**) *Left*: bright field image of the Nile Red nanosheet, (**a**) *right*: fluorescence image of the Nile Red nanosheet (Exitation filter 560 nm, Emmision Filter 620 nm). (**b**) Images of fluorescence from the Nile Red nanosheet implanted onto the wound of a mouse on days 7, 14, and 21 after implantation (representative images). More than 40% of the nanosheet remained even on day 14. About 90% of the nanosheet had disappeared on day 21.

A nanosheet alone was attached to the skin wound, more than 40% of the nanosheet remained on the ulcer for 14 days (Fig. 4).

### In vivo assessment of wound healing

For investigation of the effect of ASC spheroids carried by the nanosheet on wound healing, the following 4 animal experimental groups were examined (Table 1):

1. No treatment after removal of skin and subcutaneous tissue (Untreated skin defect)
2. No treatment for mitomycin C-induced refractory ulcer (Untreated RU)
3. Treatment by the nanosheet without ASC spheroids for mitomycin C-induced refractory ulcer (NS alone for RU)
4. Treatment by the nanosheet carrying ASC spheroids for mitomycin C-induced refractory ulcer (ASC spheroids NS for RU).

**Table 1:** Experimental animal groups for evaluation of wound healing.

Groups	N	Treatment		
		Mitomycin C^e^	Spheroids^f^	Nanosheet^g^
1. Untreated skin defect^a^	8	−	−	−
2. Untreated RU^b^	14	+	−	−
3. NS alone for RU^c^	8	+	−	+
4. ASCs spheroids NS for RU^d^	14	+	+	+

^a^No treatment after removal of skin and subcutaneous tissue.

^b^No treatment for mitomycin C-induced refractory ulcer.

^c^Treatment by the nanosheet without ASC spheroids for mitomycin C-induced refractory ulcer.

^d^Treatment by the nanosheet carrying ASC spheroids for mitomycin C-induced refractory ulcer.

^e^Topical application of mitomycin C onto the wound.

^f^Transplantation of 2 × 10^2^ spheroids onto the wound.

^g^Application of a nanosheet onto the wound.

**Figure 5:**
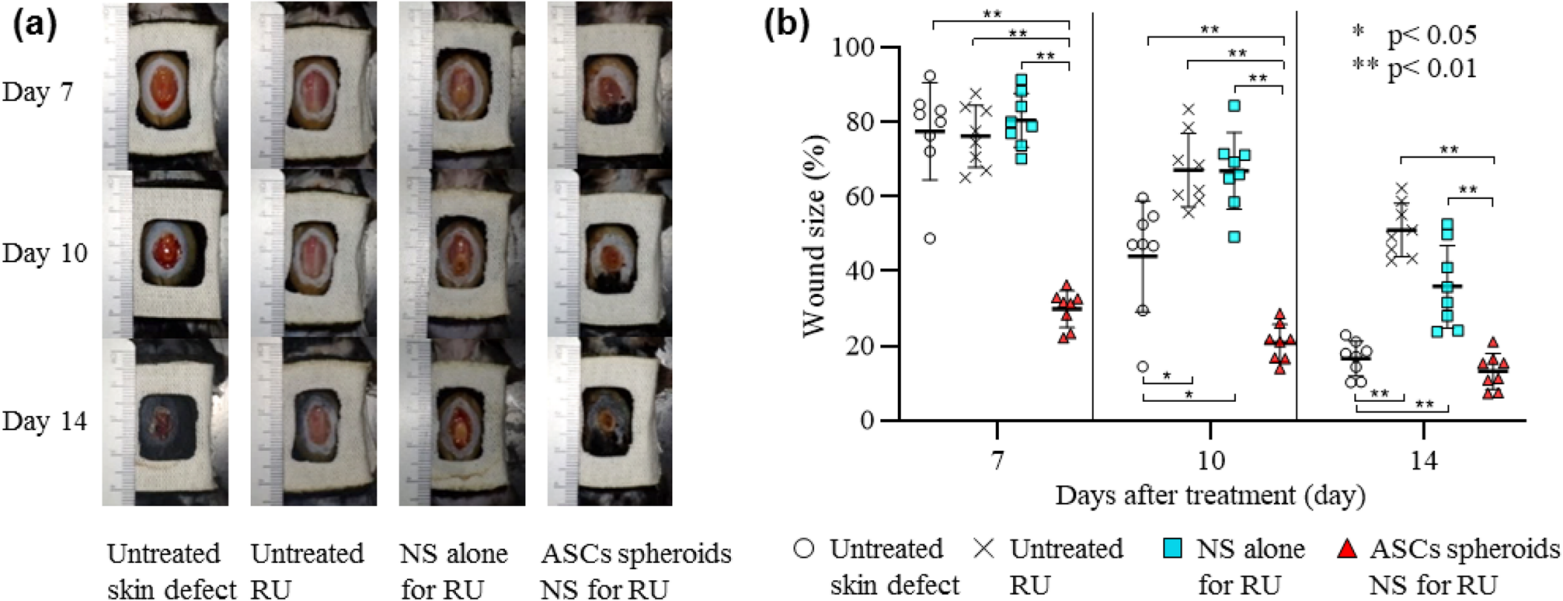
Macroscopic evaluation of wound healing. (**a**) Gross images of wounds on days 7, 10, and 14 after each treatment. (**b**) Comparison of wound sizes. The wound size on day 0 was defined as 100%. The wound size on day 14 in the animal group of ASC spheroids NS for RU is about 4-times smaller than that in the Untreated RU group (12.8 ± 4.7% vs. 49.1 ± 7.89%, *p* < 0.01).

Wound healing of the refractory ulcer was significantly promoted by ASC spheroids carried by the nanosheet. As shown in Fig. 5a, the wound size of animals in the ASC spheroids NS for RU group was significantly smaller than the sizes in the other animal groups having refractory ulcers. The wound size in the ASC spheroids NS for RU group was almost the same as that in Untreated RU group on day 3 (89.6 ± 6.4% vs. 89.5 ± 6.1%, *p* > 0.05) (Fig. 5b). Thereafter, the wound sizes in the ASC spheroids NS for RU group became significantly smaller than those in the Untreated RU group on day 7 (47.3 ± 8.5% vs. 74.6 ± 7.1%, *p* < 0.01), day 10 (24.1 ± 10.5% vs. 66.5 ± 9.4%, *p* < 0.01), and day 14 (12.8 ± 4.7% vs. 49.1 ± 7.9%, *p* < 0.01).

Additionally, the speed of wound healing in animals treated with ASC spheroids carried by the nanosheet was faster than that in animals with a simple skin defect. Thus, ASC spheroids carried by the nanosheet accelerated epithelization and/or wound shrinking.

**Figure 6:**
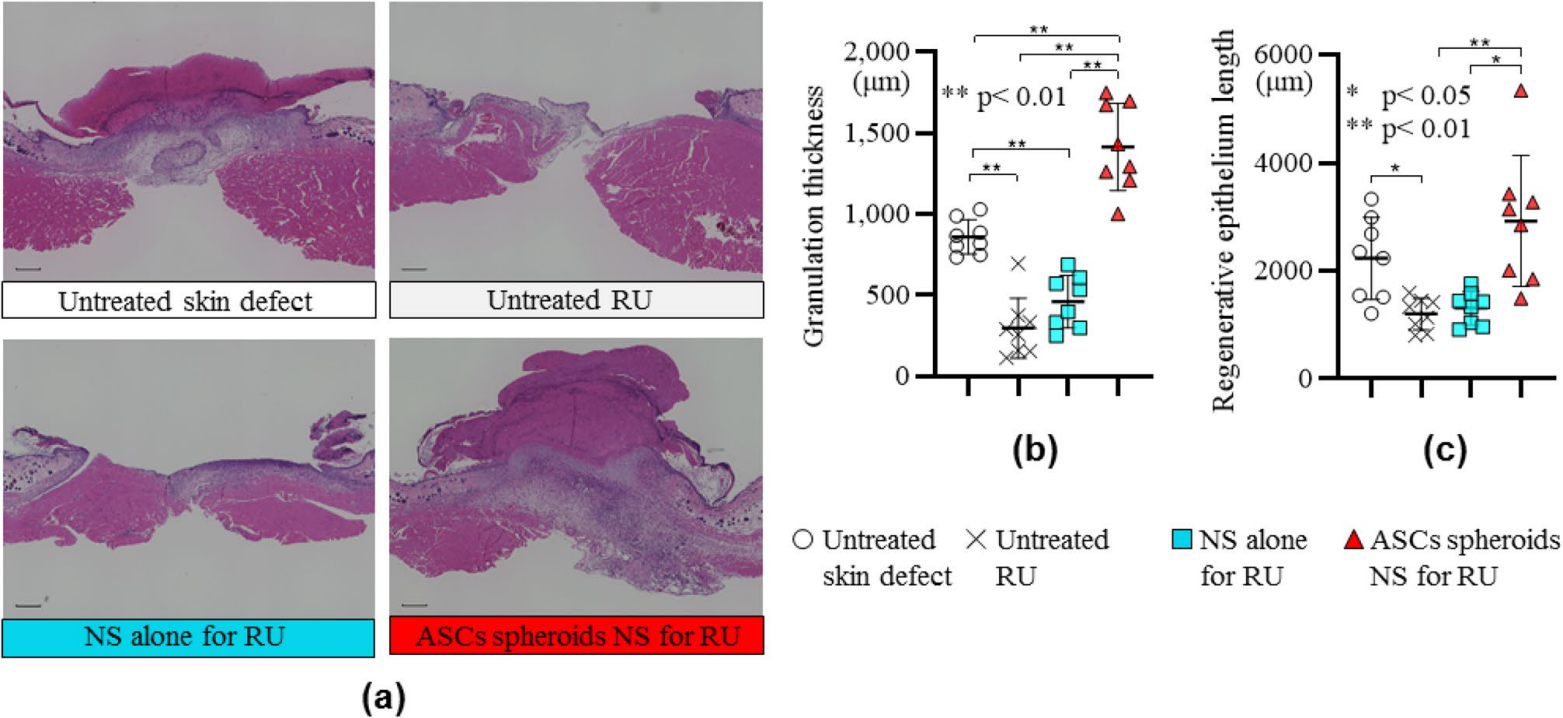
Histological evaluation of wound healing. (**a**) Pathological images of the wound on day 14 (HE stain, scale bar = 500 μm). (**b**) Thickness of formed granulation tissue in each treated group on day 14. The granulation tissue in the animal group of ASC spheroids NS for RU is about 4.8-times thicker than that in the Untreated RU group (granulation thickness: 1.41 ± 0.27 vs. 0.29 ± 0.18 mm, *p* < 0.01). (**c**) Length of regenerative epithelium in each treated group. The regenerative epithelium in the animal group of the ASC spheroids NS for RU is about 2-times longer than that in the Untreated RU group (regenerative epithelial length: 2.92 ± 1.21 mm vs. 1.12 ± 0.30 mm, *p* < 0.01).

Histological examination showed that the proliferation of granulation tissues in the wound and regeneration of the epithelium were enhanced by ASC spheroids carried by the nanosheet (Fig. 6a). The granulation tissue thickness of the animals in ASC spheroids NS for RU group was 4.8-times larger than that of the animals in the Untreated RU group and 1.6-times larger than that of the animals in the untreated skin defect group (Fig. 6b). The nanosheet without ASC spheroids had no proliferative effect on the granulation tissue of the wound (Fig. 6b). The regenerative epithelium length of the animals in the ASC spheroids NS for RU group was 2.4-times greater than that of the animals in the Untreated RU group and 1.3-times greater than that of the animals in the untreated skin defect group (Fig. 6c). The nanosheet without ASC spheroids had no effect on epithelization regeneration of the wound (Fig. 6c).

**Figure 7:**
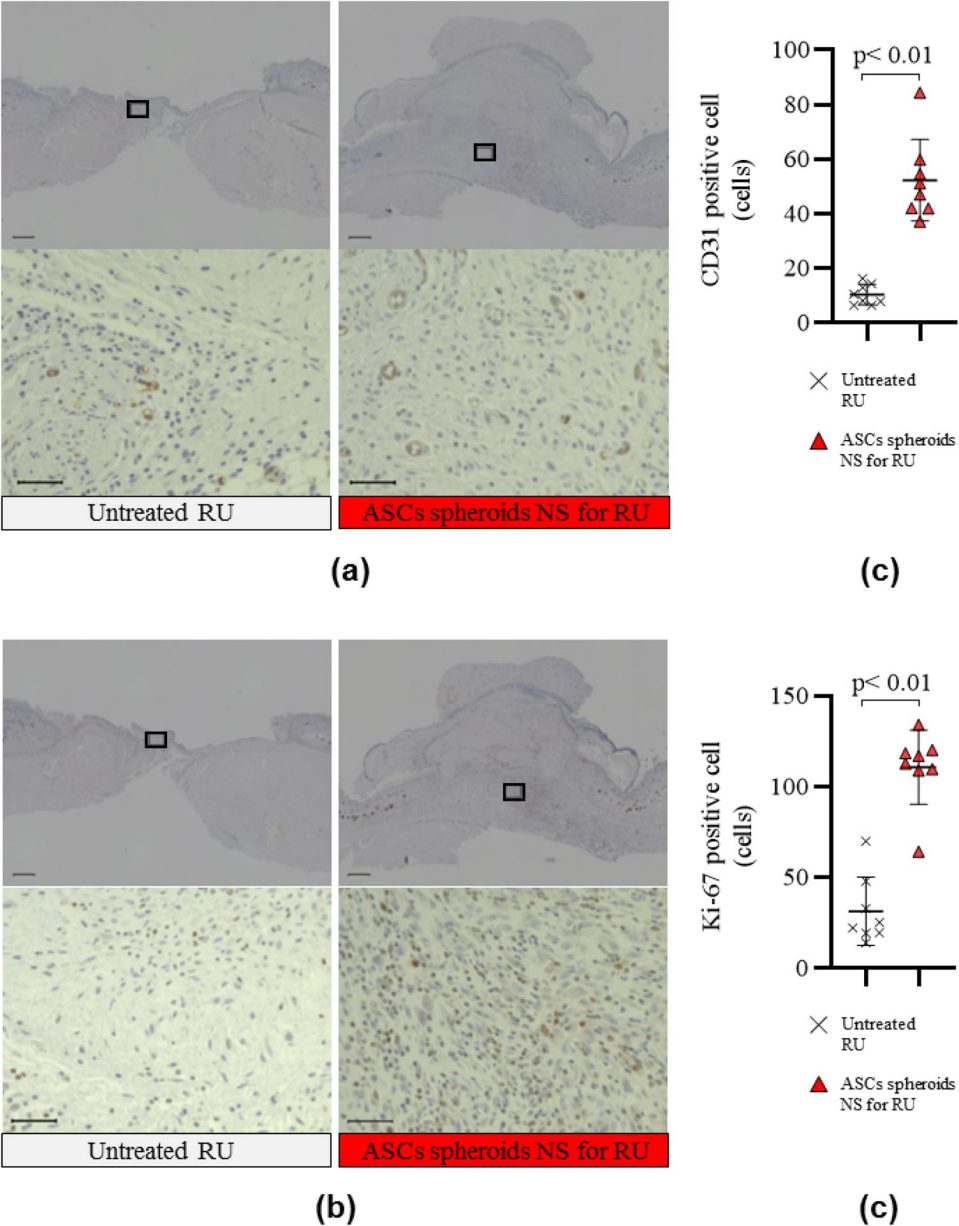
Immunohistological examination using CD31 and Ki-67 antibodies. Angiogenesis (CD31) (**a**) and cell proliferation (Ki-67) (**b**) in the skin wound were analyzed immunohistologically (representative images). Photographs shown in the lower panel are enlarged images of black boxes in the upper panels. Scale bars in the upper panels and lower panels are 500 μm and 50 μm, respectively. Numbers of CD31-positive cells (**c**) and Ki-67-positive cells (**d**).

In the wound healing process of a refractory ulcer, angiogenesis and cell proliferation were also enhanced by the ASC spheroids nanosheet (Fig. 7). Immunohistopathological examination revealed that the number of CD31-positive cells (suggesting angiogenesis) and Ki-67-positive cells (indicating cell proliferation) in the wound of the animals in the ASC spheroids NS for RU group were 1.7-times and 2.5-times, respectively, than those of the animals in the Untreated RU group.

**Figure 8:**
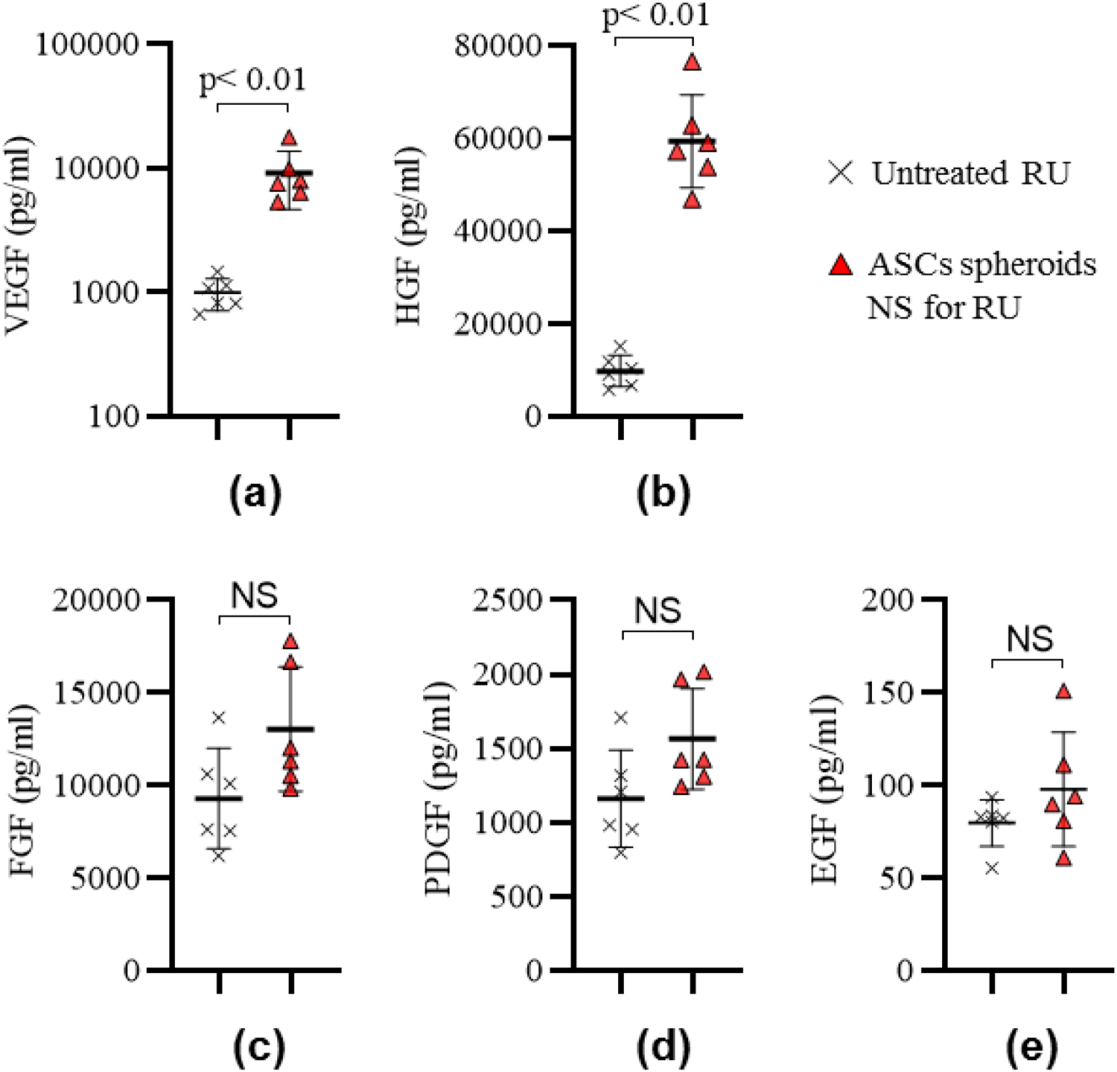
Growth factors expressed in granulation tissues on day 7. (**a**) VEGF, (**b**) HGF, (**c**) FGF, (**d**) PDGF, and (**e**) EGF (n = 6, in each). *p* < 0.01 or NS (not significant; *p* > 0).

Five growth factors secreted from ASC spheroids in granulation tissues on day 7 were measured by enzyme-linked immunosorbent assay (ELISA) (Fig. 8). The concentrations of vascular endothelial growth factor (VEGF) and hepatocyte growth factor (HGF) in the tissues in the ASC spheroids NS for RU group were approximately 9-times and 6-times larger, respectively, than those in the tissues in the Untreated RU group. The concentrations of fibroblast growth factor (FGF), platelet-derived growth factor (PDGF) and epidermal growth factor (EGF) showed no significant differences between the ASC spheroids NS for RU group and Untreated RU group.

## Discussion

This study showed that a cell transplantation method using a nanosheet extends the lifespan of ASC spheroids for more than 10 days. ASC spheroids carried by the nanosheet promoted wound healing accompanied by enhanced secretion of VEGF and HGF, suggesting paracrine effects.

This study showed that simply pipetted ASC spheroids disappeared in a few days (Fig. 2b), suggesting weak adhesion between the ASC spheroids and the skin wound. Hence, we considered that it is difficult for transplantation of ASC spheroids to be robustly engrafted onto a skin wound without a scaffold. Previous reports also suggest that the retention and function of cellular spheroids were enhanced when the spheroids were supported by several materials (coatings^17^, hydrogels^18, 19^, other materials^20, 21^). Therefore, in this study, we used a nanosheet that we fabricated as a scaffold^27^.

Our nanosheet is a polymeric ultrathin film (120 nm in thickness) made from poly(d, l, lactic acid). Nanosheets have unique mechanical properties of flexibility and physical adhesiveness due to a huge size-aspect ratio between thickness and surface area (over 10^5^; 1.2 × 10^–5^ cm in thickness, 4 × 10^0^ cm^2^ in area)^28, 29^. Considering the prolonged cellular survival of ASC spheroids carried by a nanosheet, the nanosheet is thought to enhance the adhesion between the ASC spheroids and the skin wound. We considered that these results were achieved by the physical properties of the nanosheet. ASC spheroids were only placed onto the center of the nanosheet, and there was an acellular region of the nanosheet. In that region, the nanosheet adhered well to the tissue because of its flexible structure (Fig. 3d,e). Due to the existence of that region, ASC spheroids carried by the nanosheet could be attached to the tissue. On the other hand, more than 80% of the nanosheet remained on day 7, and the luminescence of nano-lantern ASCs, which was observed on day 1, remained for 10 days. Although about 40% of the nanosheet remained on day 14, the luminescence of nano-lantern ASCs was significantly decreased on day 14 (1% luminescence compared to that on day 10). The results indicate that a nanosheet has a positive effect on retaining cells in the wound and reducing adverse effects of the external environment on the cells up to at least 10 days.

The long-term (~ 10 days) fate of therapeutic cells in wounds has not been completely elucidated^30^. In most of the studies using a skin defect animal model, a long-term (~ 10 days) tracking of the fate of ASCs (or mesenchymal stem cells) could not be performed. The exact reason is unknown. It may be because the assessment method is difficult or it may be due to immunological rejection. However, judging from various previous studies, the survival period of ASCs by topical administration is thought to be 8 days at most^31–33^. In this study, we established a noninvasive cell tracking system that can measure the viability of ASC spheroids quantitatively by using the technique of luciferase gene infection.

Owing to the establishment of BLI system, we were able to prove that the survival time of nano-lantern ASC spheroids when carried by the nanosheet was much longer than that of nano-lantern ASC spheroids alone. This imaging technique also revealed that the intensity of luminescence in nano-lantern ASC spheroids carried by the nanosheet on day 7 was increased by 1000% compared to that on day 1, suggesting that the cells of spheroids proliferated after transplantation. In addition, the luminescence was continuously observed at the site where nano-lantern ASC spheroid had been transplanted, indicating that the nanosheet can control the localization of nano-lantern ASC spheroids.

The present study showed that ASC spheroids carried by the nanosheet promoted wound healing accompanied by enhanced secretion of VEGF and HGF. The results strongly suggest that the wound healing is caused by a paracrine effect^34, 35^, in which growth factors and cytokines are released from the transplanted stem cells at the injury site.

ASC spheroids are known to produce several growth factors^36^ including VEGF, HGF, FGF, EGF, and PDGF. In this study, enhanced secretion of VEGF and HGF was seen in ASC spheroids carried by the nanosheet, but enhanced secretion of FGF, EGF and PDGF were not seen. The reason why there were no significant differences in the levels of FGF, EGF and PDGF might be because we did not detect these growth factors due to the infinitesimal amounts of the growth factors or there were really no significant differences. Since VEGF and HGF have been reported to promote proliferation/migration of vascular endothelial cells^37, 38^, epithelial cells, endothelial cells and hepatocytes^39^, the pathological responses of cells and tissues seen in this study may be explained by the actions of these growth factors.

In summary, the results of this study suggest that transplanted ASC spheroids tightly adhered to the wound by the support of the nanosheet, thus resulting in the promotion of granulation tissue formation, epithelium regeneration and angiogenesis via enhanced/prolonged secretion of VEGF and HGF.

The limitation of this study is that the optimal number of spheroids for transplantation was not investigated. Considering the limitation of repeating cell passages, 2 × 10^2^ spheroids was almost the maximum number of spheroids that we could obtain in this study. Although we obtained significant results by using this number of spheroids, the rationale for using 2 × 10^2^ spheroids is still poor and needs further investigation. There is a possibility that our method is limited for application to clinical practice because skin wounds in clinical settings are generally much larger than that in this study. Although we can enlarge the surface area of a nanosheet by using a roll-to-roll process, we need to investigate whether our transplantation method can be used for a large wound for clinical application. In addition, release of exudate from the lesion should be considered. To this end, we will use a large-scale nanosheet with a semipermeable structure^40^, which is more suitable for carrying and transplanting a large number of spheroids for clinical application.

ASC spheroids carried by the nanosheet promoted wound healing by granulation formation and angiogenesis, probably due to the enhancement of secretion of VEGF and HGF. The results suggest that ASC spheroids carried by the nanosheet can be used for treatment of skin/subcutaneous tissue resulting from various diseases such as critical limb ischemia. Additionally, our previous studies showed that medicinal ingredients (e.g., silver nanoparticles^41^ and tetracycline^42^) can be easily carried inside a nanosheet. By using this scheme, further enhancement of wound healing is expected. For example, an electroconductive nanomaterial, such as graphene, would be embedded in the nanosheet, which might enhance the wound healing process by transplanted cells^43^.

## Methods

### Nanosheet synthesis

#### Polymeric nanosheet

A biodegradable polymeric nanosheet was made by the gravure coating method, which is a roll-to-roll process using a Micro Gravure™ coater ML-120 (Yasui Seiki Co., Ltd., Kanagawa, Japan). A polyvinyl alcohol (PVA) solution (20 mg/mL) was coated on a polyethylene terephthalate (PET) film to detach the nanosheet from the substrate (line speed: 1.3 m/min, rotation speed: 30 rpm, 100 °C). Subsequently a polymer solution, in which poly(D,L-lactic acid) (*M*_w_: 180,000–530,000)^22^ (Polysciences, Warrington, PA) was dissolved in ethyl acetate (20 mg/ml), was coated on the PVA-coated PET film (line speed: 1.3 m/min, rotation speed: 30 rpm, 60 °C). The nanosheets were sterilized with ethylene oxide gas before use. The nanosheet supported on the PVA-coated PET film was cut into 2 cm × 2 cm pieces and soaked in water to dissolve the sacrificial PVA layer to obtain free-standing nanosheets.

#### Bar coating

Another PDLLA thin film with a larger thickness than that of the PDLLA nanosheet was prepared by a bar-coating method using a desktop coater (TC-3, Mitsui Electric Co., Ltd, Chiba, Japan). A PDLLA solution (10 wt% in ethyl acetate) was coated on the PVA-coated PET film using the desktop coater (bar: OSP-100, speed: 10 mm/min). After coating, the PDLLA thin film was dried on a hot plate at 50 °C for 1 h.

#### Nile Red nanosheet

The nanosheet after being implanted onto the skin wound cannot be confirmed by naked eyes owing to its transparency. Thus, for the confirmation and investigation of the adhesion state of the nanosheet after being implanted onto the skin wound, we made another type of nanosheet that contained a fluorescence dye.

For the fluorescent dye, Nile Red (Tokyo Chemical Industry, Tokyo, Japan) was dissolved in the polymer solution (final concentration of 3.5% (w/v)), and the Nile Red nanosheet was prepared by the same procedure as above.

### Characterization of PDLLA nanosheets

The thickness and height image of the nanosheet was characterized by AFM (Innova-AFM, Bruker, Tokyo, Japan). The tack-separation test was conducted to evaluate the adhesive property of PDLLA nanosheets on chicken muscle by exploiting a tensile tester (EZ-SX, Shimadzu, Kyoto, Japan) as previously reported^26^. In brief, a PDLLA sheet supported by a 3D-printed plastic frame made of ABS was attached to the surface of chicken breast muscle (1 cm × 1 cm × 0.5 cm). The PDLLA thin film was kept attached to the chicken surface for 30 min at room temperature and the plastic frame was lifted (5 N load cell, 10 mm min^−1^) until the film was detached from the surface, from which force–stroke curve was recorded for different thickness of the films.

### Nano-lantern infection into ASCs

Adipose derived mesenchymal stem cells (ASCs; 6 passages at the time of purchase) from C57BL/6 mice were purchased from Cyagen Biosciences (Santa Clara, CA) and were used in the experiments. The cells were cultured in Dulbecco’s modified Eagle’s medium containing nutrient mixture F-12, 10% fetal bovine serum, and 1% penicillin streptomycin under the conditions of 5% CO_2_, 37 °C, and atmospheric pressure. Cells within 10 passages were used in the following experiments.

For nano-lantern expression, a retrovirus expressing a gene encoding the luminescent protein nano-lantern^44^ was used. Nano-lantern was expressed together with a puromycin resistance gene via an internal ribosome entry site. A culture dish was coated with 80 μg/ml recombinant human fibronectin fragment (RetroNectin^®^; Takara Bio, Tokyo, Japan). ASCs were then plated in the coated dish, and this allowed nano-lantern to be introduced into ASCs (so-called the supernatant method). The medium was changed 24 h after the infection. Six μg/ml of puromycin was added to the medium in order to select the cells expressing nano-lantern.

### Measurement of surface antigens expressed on the cellular membrane

To confirm no difference between surface antigens expressed on the cellular membrane of ASCs and nano-lantern ASCs, flowcytometric analysis was carried out.

At first, Fc block treatment was performed before antibody staining. The cell suspension was then incubated for 30 min at 4 °C with PE hamster anti-mouse CD29, Alexa Fluor 647 conjugated rat anti-mouse CD34, BV480 rat anti-mouse CD44, PerCP-Cy5.5 rat anti-mouse CD45, PerCP-Cy5.5 rat anti-mouse 7AAD, APC-Cy7 mouse anti-rat CD90/mouse CD90.1, BV421 rat anti-mouse CD105, and PE-Cy7 rat anti-mouse Ly-6A/E (Sca-1) (all from BD Bioscience). After washing the cells twice with PBS, they were analyzed by FACS Canto II (BD Biosciences). The data obtained were analyzed by FlowJo™ software (BD Biosciences).

### Preparation of ASC spheroids carried by the nanosheet

A small amount of the cell suspension (including ~ 1.0 × 10^3^ cells) was pipetted into a well of a 96-well low adsorption plate (PrimeSurface^®^; MS-9096U, Sumitomo Bakelite, Tokyo, Japan) and this operation was repeated 192 times. Incubation for 24 h allowed the cells in each well to aggregate and to form a spheroid (Fig. 1a), and a total of 192 spheroids were obtained.

All of the spheroids (~ 2 × 10^2^) were collected into a 50 ml conical tube. After centrifugation at 1200 g for 2 min, the supernatant was removed. Hanks’ balanced salt solution (20 μl) was added to the spheroids pellet and gently pipetted so as not to discrete the spheroids. A concentrated suspension of 2 × 10^2^ spheroids was gently dropped onto the center of a 3 × 3 cm nanosheet (Fig. 1b). The dropped spheroids stayed within a narrow region (~ 2 mm range) of the nanosheet.

### Confirmation of a correlation between intensity of luminescence and number of spheroids

Spheroids with a number from 0 to 384 were transferred to each well with 200 μl medium using a 24-well plate (Supplementary Fig. 1). The luminescent molecule Coelenterazine h (Fujifilm Wako Chemicals U.S.A, Richmond, VA) was added to each well at 10 μg. The intensity of luminescence was measured by IVIS (Parkin Elmer).

### Animals

Eight-week-old male C57BL/6 mice (25–30 g) were purchased from Japan SLC (Shizuoka, Japan). A mixture of 3 anesthetics (midazolam at 0.3 mg/kg, medetomidine at 4 mg/kg, and butorphanol at 5 mg/kg) were intraperitoneally administered, and the following treatment was performed. The back of each mouse was shaved using shaving cream (Epilat^®^; Kracie Home Products, Tokyo, Japan). A skin defect wound including the panniculus carnosus was created with an 8 mm punch on the back of the mouse (Fig. 1). In order to create an intractable skin ulcer, 20 μl of 1 mg/ml of mitomycin C (10 v/v% ethanol and 90 v/v% ethilene glycol) (Wako Pure Chemical Industries, Osaka, Japan) was topically applied to spread over the entire wound and the mouse was left calmly for 10 min. After washing the wound with saline, the wound was covered with several dressings as described below (as shown in Fig. 1c). A hydrocolloid dressing (40 × 30 mm, DuoactiveET^®^; ConvaTec, Berkshire, UK) with a 10-mm diameter hole was attached to the back of each mouse. After that, in the case of using a nanosheet without ASC spheroids, the nanosheet was attached to the wound. In the case of using a nanosheet carrying ASC spheroids, the nanosheet was attached to the wound so that the spheroids were in contact with the wound surface. Next, ethylene/propylene rubber (Eptsealer^®^; Nitto Denko, Osaka, Japan) was applied to protect the wound. To prevent wound contraction, wound dressings were sutured at 9 different points with a 5-0 nylon in a circumferential manner. Film dressing (Opsite^®^; Smith and Nephew plc, Watlford, UK) was applied to the small window to prevent dryness. Finally, the mouse trunk and wound dressings were fixed with adhesive tape (Silkytex^®^, ALCARE Co, Tokyo, Japan).

For observation of the wound state, the film dressing and adhesive tape were removed and replaced with new ones after the observation. The wound state could be observed through the small window (Fig. 1d). All animal experiments were carried out in accordance with the ARRIVE guidelines, and the study was approved by the animal Care and Use Committee of the National Defense Medical College, Saitama, Japan (permission number: 19013).

### Evaluation of wound healing

#### Measurement of wound size

To measure the wound size, the wound was photographed with a fixed distance between the animal and camera on days 0, 3, 7, 10, and 14 after transplantation. The wound area on day 0 was defined as 100%. The wound size was analyzed with Image J software^45^.

#### Visualization of luminescence from ASC spheroids by BLI

For in vivo visualization of the luminescence from ASC spheroids, 100 μl coelenterazine h solution (0.5 mg/ml) was intravenously administered to the animals in which ASC spheroids had been transplanted. ASC spheroids were transplanted with or without a nanosheet. When transplanting ASC spheroids without a nanosheet, ASC spheroids with 20 ul HBSS were directly pipetted onto the wound. After that, the wound was covered with adhesive tape in the same manner as that shown in Fig. 1d,e. The luminescence was visualized using IVIS Lumina series III (PerkinElmer) and the total amount of luminescence was calculated using Living Image^®^ software 3.0 (PerkinElmer).

#### Observation of the fate of the nanosheet on the wound

Transition of the adhesion state of the nanosheet on the wound was observed using a nanosheet containing Nile Red as described above (S1.2). A refractory skin ulcer (same as that for Untreated RU) was made and the Nile Red nanosheet was attached onto the wound. Fluorescence from Nile Red (Ex. 560 nm, Em. 620 nm) was visualized by IVIS on days 3, 7, 14, and 21 to evaluate the extent of the remaining nanosheet.

#### Histological analysis

After photographing the wound on day 14, skin tissue including the wound area was excised for pathological examination. The specimens were fixed in 10% formalin for 24 h. The sections were processed routinely and stained with hematoxylin and eosin (HE). By microscopic examination using the HE-stained samples, the thickness of the granulation tissue and the regeneration of the epithelium at the skin defect were measured.

As well as HE staining, some of the specimens obtained from animals in two groups (Untreated RU and ASCs spheroids NS for RU groups) were used for immunohisitological examination using CD31 (DIA-300; DIANOVA GimbH, Hamburg, Germany) and Ki-67 (NCL-Ki67p; Leica Biosystems, Newcastle, UK) antibodies. Briefly, for both stains, antigen retrieval was performed in paraffin-embedded sections with 10 mM citrate buffer for 10 min. Thereafter, 3% H_2_O_2_ was used to inactivate endogenous peroxidase, and Blocking One (Nacalai Tesque, Kyoto, Japan) was used to block nonspecific binding reactions. The sections were then incubated with primary and secondary antibodies. The sections were treated with 3, 3′‐diaminobenzidine‐4HCl (DAB) and Mayer's hematoxylin nuclear counterstain as per the usual protocols. Microscopic images were taken of five random fields from each slide at high power fields (×400). The positive cells were counted and the average value was calculated.

#### Measurement of levels of growth factors

Levels of growth factors expressed in the granulation tissues were measured. Granulation tissues of the wound were collected on day 7. Five hundred μl of RIPA lysis and extraction buffer and 5 μl Halt protease inhibitor cocktail (Thermo Fisher Scientific) were added to the granulation tissues. The granulation tissues were cut into small pieces with scissors and were incubated on ice for 30 min. Thereafter, specimens were adequately homogenized by a Physcotron homogenizer (Microtec) with 30,000 rpm within a short time (30–45 s). After homogenization, the specimens were additionally incubated on ice for 30 min and centrifuged twice. Using the supernatant of the specimen, EGF, VEGF, FGF, PDGF and HGF levels were measured by a Mouse Quantikine ELISA Kit (R&D Systems). A small amount of the supernatant was used for measurement of protein concentration using a Pierce™ BCA protein assay kit (Thermo Fisher Scientific).The data were analyzed by creating a 4-parameter logistic curve with Image J software.

## Supplementary information﻿

Supplementary Informations.

## Data Availability

All data generated during and/or analyzed during the current study are available from the corresponding author on reasonable request.

## Abbreviations

ASCs: Adipose-derived mesenchymal stem cells
ECM: Extracellular matrix
PDLLA: Poly(d, l-lactic acid)
BLI: Bioluminescence imaging
ELISA: Enzyme-linked immunosorbent assay
VEGF: Vascular endothelial growth factor
HGF: Hepatocyte growth factor
FGF: Fibroblast growth factor
PDGF: Platelet-derived growth factor
EGF: Epidermal growth factor
