# Supplementary material for: Enhanced cellular engraftment of adipose-derived mesenchymal stem cell spheroids by using nanosheets as scaffolds
Source: Sci Rep. 2021 Jul 14;11:14500. doi: 10.1038/s41598-021-93642-6 (PMC8280158; doi:10.1038/s41598-021-93642-6)
Supplement: Supplementary file 1 — Supplementary Informations. [file 41598_2021_93642_MOESM1_ESM.docx]

**Title**

Enhanced Cellular Engraftment of Adipose-derived Mesenchymal Stem Cell Spheroids by Using Nanosheets as Scaffolds

**Authors**

Hisato Nagano^1^, M.D., Yoshitaka Suematsu^2^, Megumi Takuma^3^, Shimpo Aoki^4^, M.D., Ph,D., Ayano Satoh^5^, Ph.D., Eiji Takayama^6^, Ph.D., Manabu Kinoshita^7^, M.D., Ph.D., Yuji Morimoto^8^, M.D., Ph.D., Shinji Takeoka^9^, Ph.D., Toshinori Fujie^3^, Ph.D., and *Tomoharu Kiyosawa^1^ M.D., Ph.D.

**Affiliation:**

^1^ Department of Plastic and Reconstructive Surgery, ^7^Department of Immunology and Microbiology, ^8^Department of Physiology, National Defense Medical College, Tokorozawa, Saitama 359-8513, Japan

^2^ Department of Life Science and Medical Bioscience, Graduate School of Advanced Science and Engineering, Waseda University, Tokyo 162-8480, Japan

^3^ School of Life Science and Technology, Tokyo Institute of Technology, Yokohama, Kanagawa 226-8501, Japan

^4^ Tissue Engineering and Wound Healing Laboratory, Division of Plastic Surgery, Brigham and Women’s Hospital, Harvard Medical School, 75 Francis Street
Boston, MA 02115 USA

^5^ Graduate School of Interdisciplinary Science and Engineering in Health Systems, Okayama University, Okayama 700-0082, Japan

^6^ Department of Oral Biochemistry, Asahi University School of Dentistry, Gifu 501-0296, Japan

^9^ Institute for Advanced Research of Biosystem Dynamics, Research Institute for Science and Engineering, Waseda University, 3-4-1 Ohkubo, Shinjuku-ku, Tokyo 169-8555, Japan

**Abbreviations:**

ASCs, adipose-derived mesenchymal stem cells; ECM, extracellular matrix; PDLLA, poly(D,L-lactic acid); BLI, bioluminescence imaging; ELISA, enzyme-linked immunosorbent assay; VEGF, vascular endothelial growth factor; HGF, hepatocyte growth factor; FGF, fibroblast growth factor; PDGF, platelet-derived growth factor; EGF, epidermal growth factor

***Corresponding Author**: Tomoharu Kiyosawa, M.D., Ph.D., Department of Plastic and Reconstructive Surgery, National Defense Medical College Hospital, Tokorozawa, Saitama 359-8513, Japan

**E-mail**: xoo@ndmc.ac.jp

**Tel**: ＋81-4-2995-1211, ext. 6162

**Fax**: ＋81-2997-5156

**SUPPLEMENTARY FIGURE**

**
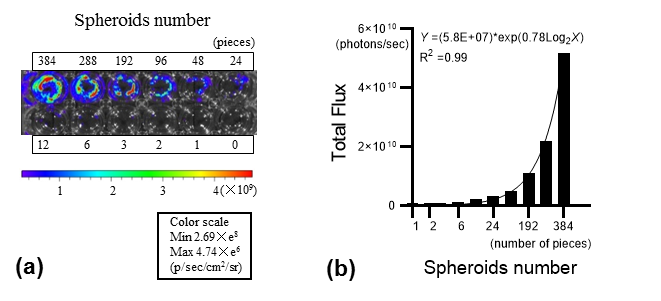
**

**Supplementary Fig. 1. Increase in luminescence in proportion to the number of spheroids**

(a) Luminescence from spheroids that consisted of nano-lantern-transfected ASCs. Each well in a 24-well plate contained spheroids with numbers of 384, 288, 192, 96, 48 and 24 in the upper row and 12, 6, 3, 2, 1 and 0 in the lower row.

(b) Intensity of luminescence (total flux) from each number of nano-lantern ASC spheroids. The luminescence intensity was correlated with the number of spheroids (R^2^ = 0.99).


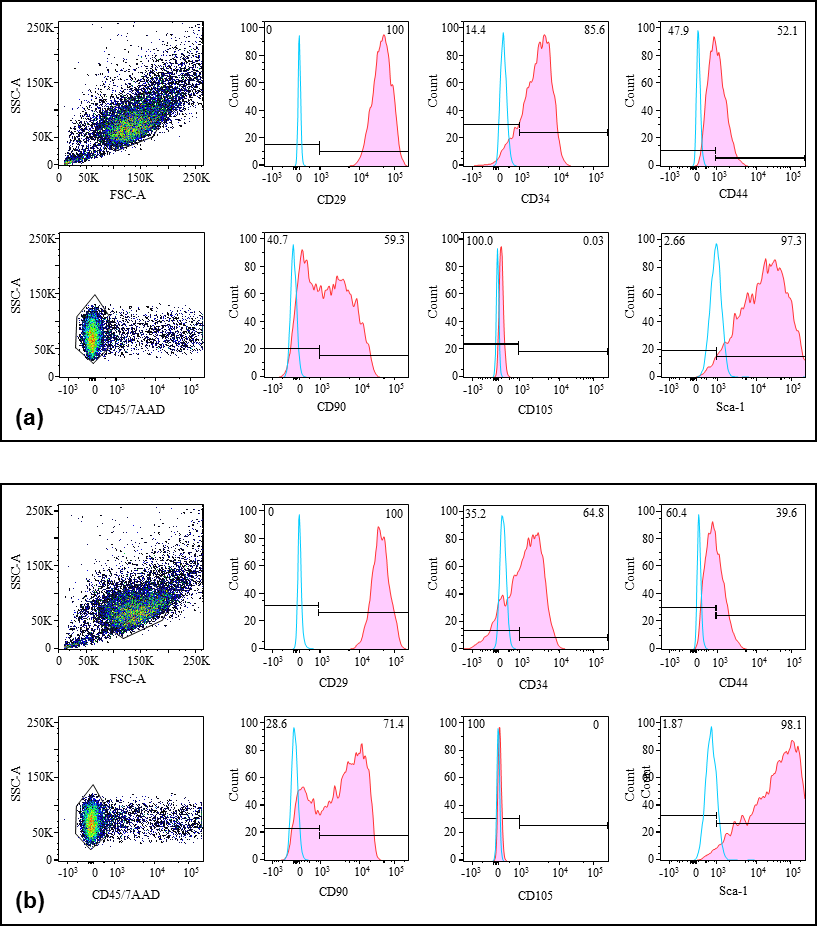


**Supplementary Fig. 2. Flowcytometric analysis of surface antigens expressed on the cellular membrane of ASCs (a) and nano-lantern ASCs (b)**

Six kinds of antibodies (CD29, CD34, CD44, CD90, CD105, Sca-1) were analyzed using two gates (singlets plot and 7AAD/CD45 negative plots shown as 2 columns on the left in box (a) and box (b). Pink-colored histograms were obtained from each sample and blue-colored histograms were obtained from each isotype control. Positive percent rate and negative percent rate are shown above each histogram. There was no significant difference between the expression levels of antigens on ASCs and those on nano-lantern ASCs.
